# Needs assessment for physical activity information during COVID-19 among a nationally representative sample of parents and children ages 6–17 in the United States: a cross-sectional study

**DOI:** 10.1186/s12889-021-12024-9

**Published:** 2021-10-28

**Authors:** Ashleigh M. Johnson, Emily Kroshus, Pooja S. Tandon

**Affiliations:** 1 Center for Child Health, Behavior, and Development, Seattle Children’s Research Institute, 1920 Terry Ave, Seattle, WA 98101 USA; 2 Department of Pediatrics, University of Washington, 4245 Roosevelt Way NE, Seattle, WA 98105 USA

**Keywords:** Youth, Physical activity/exercise, Social support, Community health promotion

## Abstract

**Background:**

The COVID-19 pandemic presented novel barriers to youth physical activity engagement. Identifying what resources parents and children are interested in receiving can support efforts to mitigate the negative impact of the pandemic on youth physical activity behavior. This study aimed to identify physical activity-related information needs during the COVID-19 pandemic among a nationally representative sample of American parents of children 6–10 years-old and parent-child dyads of children 11–17 years-old.

**Methods:**

A cross-sectional survey was conducted by a market research company in October–November 2020. Parents and children were asked about their interest in specific types of information about helping their family and themselves, respectively, be active (Yes/No). Weighted percentages were calculated for reported information needs and compared using two-sample test of proportions.

**Results:**

Final analytic sample was 1000 parents (55.4% female; 74.7% White; 74.0% non-Hispanic); 500 children 11–17 years-old (52.1% male; 77.6% White). Over 40% of participants were interested in information about being active during COVID-19. Parents were more likely to be interested in information if they always (versus never) worked from home [53.3% (95% CI: 43.3–63.0%) versus 22.0% (95% CI: 14.9–31.3%), *p* < 0.001]; had children attending school remotely versus in-person [47.3% (95% CI:40.2–54.5%) versus 27.5% (95% CI: 19.6–37.1%), *p* < 0.001]; and lived in a big city versus a rural area [66.5% (95% CI:54.5–76.7%) versus 34.1% (95% CI: 22.8–47.6%), *p <* 0.001]. Children most interested were those who did not have resources for online activity engagement and those worried about their safety or getting infected with COVID-19. Children were also more likely to be interested if their parents worked full-time versus not working [48.6% (95% CI:41.7–55.6%) versus 31.5% (95% CI: 24.1–39.9%), *p* < 0.001], and lived in a big city versus a rural area [57.2% (95% CI:45.3–68.3%) versus 27.8% (95% CI:17.8–40.7%), *p <* 0.001].

**Conclusions:**

Families are interested in physical activity resources, particularly those whose daily routines and opportunities for physical activity may have been most significantly impacted by the pandemic. This includes parents who always worked from home or whose children attended school remotely. Identifying felt needs is an important step in developing tailored interventions that aim to effectively and sustainably support families in promoting physical activity.

## Background

The physical and mental health benefits of physical activity participation are well-documented [1–4], yet activity levels among children remain low [5]. Determinants of physical activity span the social ecological spectrum, and can include individual, family, organizational (i.e., school), community, and cultural influences [4, 6]. The COVID-19 pandemic and resulting restrictions presented novel barriers to physical activity engagement, including disruptions to the school environment and other organized physical activity opportunities, resulting in a decline in youth physical activity levels, [7, 8] as well as the documented decrease in cardiorespiratory fitness levels due to such pandemic-related mitigation measures [9]. Such disruptions likely disproportionately impacted children experiencing other pre-existing physical activity barriers. For example, although youth of low (versus high) socioeconomic status (SES) typically report lower physical activity levels, they have higher levels of active commuting to school [10–12], and report more in-school physical activity [13]. Additionally, rural youth are less likely than urban youth to meet physical activity guidelines, [14] likely due to the cultural and structural limitations specific to rural areas (e.g., limited resources and fewer outlets for physical activity) [15]. Thus, the transition to remote learning during the pandemic may have disproportionately impacted children of lower SES and rural youth that have fewer physical activity opportunities outside of school [15, 16]. It is therefore important to examine how to best support families, schools, and communities in efforts to mitigate the secondary impact of large-scale disruptions to in-person learning, such as those presented by the COVID-19 pandemic, on children’s activity levels.

Parents, in particular, can have a strong influence on their children’s physical activity behavior. This role was of heightened importance during the COVID-19 pandemic as children spent more time at home and had decreased access to other opportunities for physical activity, such as physical education (PE) class and organized sports. Parent support for child activity, both tangible (e.g., transportation, instrumental) and intangible (e.g., motivation, encouragement), is positively associated with child physical activity [17–19]. However, during the pandemic, parents’ capacity to support children’s physical activity was likely affected by factors such as work and school status, and other pandemic-related stressors. Prior evidence from pre-pandemic suggests that cognitive and motivational factors, such as perceived capability, intention, and perceived importance of physical activity, influence parent support for child physical activity [20, 21]. Such factors may have been influenced by structural constraints and stressors related to the COVID-19 pandemic [22], and affected caregivers’ ability to provide support [23]. Understanding of how to best support parents during the pandemic can be informed, in part, by determining their felt needs around youth physical activity through an informational needs assessment.

Another important consideration is that the role of parents in facilitating youth physical activity changes as their children age and become more independent. Among younger children, parents influence child physical activity through direct involvement, and modeling of activity plays an important role in establishing social norms around physical activity [21, 24–26]. However, there may be added stress on parents of younger children as pandemic restrictions and closures negatively impacted their ability to support their child’s activity, and many tried to balance their work with increased responsibilities related to children attending school remotely. Among older children, parental modeling wanes as peers become a stronger influence [21, 25], and adolescents’ physical activity attitudes and competence become important predictors of their behavior [26]. Thus, providing older children with appropriate resources and information has the potential to support positive attitudes around physical activity, and can help them feel competent in their ability to be physically active [4]. Still, parents of older children may have had a larger role in supporting their child’s physical activity during the pandemic compared to pre-pandemic, as closures and restrictions limited many physical activity opportunities outside the home (e.g., playing with friends, organized sport). Relevant information and resources, as identified via an information needs assessment, can support parents as facilitators of their child’s physical activity and support older youth as they practice more control of their physical activity behavior, both during and post-pandemic.

Prior studies about parent physical activity-related information needs have most often been examined among parents whose child is experiencing a long-term or chronic condition, and less is known about parental preferences around promoting family physical activity at the family level, particularly during the COVID-19 pandemic. Still, physical activity has been identified by parents as a priority in their child’s health and well-being [27], and a majority of parents seek online health information for both themselves and their children [28]. An important component of effective, well-developed behavioral interventions is the use of a needs assessment, including examination of perspectives from members of the intended community [29]. However, little is known about what information, materials, or resources would be helpful to parents and children in the context of behavioral interventions targeted at promoting activity during the pandemic, which resulted in widespread disruption to families’ daily life. Identifying felt needs is an important first step in developing intervention approaches that take parent and child preferences into account and would have relevance even beyond the pandemic. Thus, the purpose of this study is to identify physical activity-related information needs during the COVID-19 pandemic among a nationally representative sample of parents of children 6–10 years-old and parent-child dyads of youth 11–17 years-old. A secondary objective was to compare parent and child information needs by parent and child gender, school and work status, parent education, and urbanicity.

## Methods

### Overview and study population

A cross-sectional survey was conducted in October–November 2020 by a market research company (YouGov) using a United States (US) nationally representative sample of 500 parents of children 6–10 years-old and 500 parent-child dyads among youth 11–17 years-old. YouGov interviewed 547 parents with children 6–10 years-old, and 535 parent-child dyads with children 11–17 years-old. Participants with children 11–17 years-old also recruited one of their children in that age range for a follow-up survey. Participants were then matched down to samples of 500 in each cohort (parents of 6 to 10-year-olds, parents of 11 to 17-year-olds; *N* = 1000) according to US census-based sampling frames. For parents of children 6–10 years-old, participants were matched to a three-way sampling frame of age, race, and education. Participants for children 11–17 years-old were matched to a four-way sampling frame of gender, age, race, and education. Both frames were constructed using stratified probability sampling from the full 2017 American Community Survey (ACS). The matched cases were weighted to sampling frames corresponding to US parents with children 6–10 or 11–17 years of age. Additionally, all 1000 cases were further weighted to a sampling frame corresponding to US parents with children 6–17 years of age using propensity scores. Details for YouGov’s sampling matching procedure can be found in Rivers (2007) [30]. Parents of children 6–10 years-old were offered the equivalent of $3 in incentives to take the survey. Parents of children 11–17 years-old were offered $3 to take the initial parent survey and an additional $8 when their child completed the survey. All study procedures were approved by an internal institutional review board (IRB).

### Measures

#### Information needs assessment

A needs assessment of physical activity information was conducted through a series of items among parent participants and children 11–17 years-old. Parent participants were asked whether they were interested in information about helping their family be physically active during the COVID-19 pandemic (Yes/No). If yes, they were then asked about the kind of information they would want, including: “*Why it’s important to be physically active during the novel coronavirus (COVID-19) pandemic”;* “*How to avoid getting the novel coronavirus (COVID-19) pandemic while doing physical activity or sports”*; “*Online resources (like videos, online classes) for your child to do physical activity*”; and “*Ideas about physical activity your child can do* 1) *alone,* 2) *inside,* and 3) *outdoors*”. Children 11–17 years-old were asked similar questions regarding their own interest in information about being physically active.

#### Child barriers to physical activity

Children 11–17 years-old also reported on barriers to physical activity in the past week (Yes/No), including, “*I don’t like doing physical activity*”; “*I don’t know what to do*”; “*It’s not safe to go outside near my house to play/be active*”; “*I don’t have anyone to play/be active with*”; “*The weather is not good for outdoor activities*”; “*I don’t have the right equipment to play/be active*”; “*I’m worried I might get COVID-19*”; and “*I want to do online physical activity but I don’t have the right resources (e.g., laptop, wifi)*”.

#### Demographics

Demographic characteristics included parent birth year; child age (6–10 years, 11–17 years), gender (male, female, non-binary), race (Caucasian or White, African-American or Black, Asian or Asian American, Native American or Alaskan native, Native Hawaiian or other Pacific Islander, other), and ethnicity (Hispanic, Mexican, or Latin American ethnicity); child school status (in-person, remote/virtual, hybrid); parent work status (full time, part-time, not working); parent work from home status (all of the time, some of the time, no); parent education (no high school, high school graduate, some college, 2-year college, 4-year college, post graduate); and household urbanicity (big city, smaller city, suburban area, small town, rural area). Parents reported on their own demographics and for children 6–10 years-old. Older children 11–17 years-old reported on their own demographics.

### Statistical analysis

Data management and statistical analyses were performed using Stata 15.1 (StataCorp, College Station, TX). Descriptive statistics were calculated for parent and child demographic characteristics. Categorical variables were presented as n’s and weighted proportions, and continuous variables were presented as means with standard deviations (SD). We calculated weighted percentages and corresponding 95% confidence intervals (CIs) for reported physical activity information needs (i.e., needs assessment). Informational needs were examined among 1) parents of children 6–10 and 11–17 years-old and 2) children 11–17 years-old. Information needs were examined overall and by parent and child characteristics. Children’s information needs were also examined by their perceived barriers to physical activity in the past week. The two-sample test of proportions was used to compare the proportion of participants that reported interest in information about being physically active during the pandemic. The proportion of participants who responded “Yes” to this survey item were compared by parent and child gender, school and work status, parent education, and urbanicity. Statistical significance was set at *p* < 0.05 for all analyses.

## Results

The final analytic sample included 1000 parents with children 6–17 years-old and 500 children 11–17 years-old. Parent participants were predominantly female (55.4%), White (74.7%), and non-Hispanic (74.0%) (Table 1). Over 62% of parents had at least some college education. Child participants were predominantly male (52.1%) and White (77.6%), with a mean age of 14.2 years (SD = 2.0).

**Table 1:** Child and Parent Participant Characteristics

CHILD (*n* = 500)		
	Mean (SD)	
Age (years)	14.2 (2.0)	
	n	% (95% CI)^a^
Gender		
Male	252	52.1 (47.0–57.2)
Female	239	45.8 (40.8–51.0)
Non-Binary	5	1.1 (0.4–2.9)
Unknown	4	0.9 (0.3–2.5)
Hispanic, Mexican, or Latin American ethnicity	113	22.6 (19.1–26.5)
Race		
Caucasian or White	395	77.6 (73.0–81.7)
African-American or Black	75	13.7 (10.5–17.6)
Asian or Asian American	21	4.4 (2.7–7.0)
Native American or Alaskan Native	18	3.7 (2.1–6.2)
Native Hawaiian or other Pacific Islander	2	0.3 (0.1–1.3)
Other races	38	8.7 (6.2–12.2)
School Status		
In-person	96	18.4 (14.8–22.6)
Remote	251	49.8 (44.7–54.9)
Hybrid	153	31.8 (27.2–36.8)
**PARENT (*n* = 1000)**		
Gender		
Male	440	44.6 (40.9–48.3)
Female	560	55.4 (51.7–59.1)
Hispanic, Latino, or Spanish Origin	204	26.0 (22.4–30.0)
Race		
Caucasian or White	781	74.7 (71.0–78.1)
African-American or Black	126	12.0 (10.0–14.3)
Asian or Asian American	26	3.7 (2.4–5.7)
Native American or Alaskan Native	39	3.8 (2.6–5.4)
Native Hawaiian or other Pacific Islander	3	0.5 (0.1–1.6)
Other races	67	9.9 (7.2–13.5)
Education		
High School Graduate or below	322	37.6 (33.9–41.5)
Some College	323	26.4 (23.6–29.4)
4-year College	224	22.5 (19.8–25.5)
Post-graduate	131	13.4 (11.2–16.1)
Current Work Status		
Work full time (> 35 h/week)	513	51.1 (47.4–54.8)
Work part-time	139	13.5 (11.1–16.3)
Not working	348	35.4 (31.8–39.1)
Working from home		
Yes, all the time	240	23.5 (20.7–26.6)
Yes, some of the time	145	14.8 (12.4–17.6)
No	267	26.3 (23.1–29.7)
Not applicable	348	35.4 (31.8–39.1)

^a^Weighted

### Parent information needs

Parent information needs were similar between parents of younger children (6–10 years-old) compared to parents of older children (11–17 years-old). For example, 39.3% (95% CI: 34.5–44.3%) of parents of younger children and 45.4% (95% CI: 40.4–50.6%) of parents of older children reported interest in information about helping their family be active during COVID-19 (Table 2). Regarding the kind of information that parents wanted, overall interest tended to be lower for why it is important to be active during COVID-19 and how to avoid getting COVID-19 while doing physical activity, and higher for online resources for their child to do physical activity and ideas about physical activity their child could do alone, inside, or outdoors.

**Table 2:** Parent Physical Activity Information Needs by Child and Parent Characteristics

		Interest in information about helping family be active during COVID-19 (% Yes)	“What kind of information would you want?” (% Yes)					
			Why it’s important to be active during COVID-19	How to avoid getting COVID-19 while doing PA	Online resources for your child to do PA	Ideas about PA your child can do…		
						…alone	…inside	…outdoors
	n	% (95% CI)	% (95% CI)	% (95% CI)	% (95% CI)	% (95% CI)	% (95% CI)	% (95% CI)
Total	1000	41.9 (38.9–45.5)	30.6 (27.4–34.1)	32.8 (29.4–36.3)	36.7 (33.2–40.3)	35.1 (31.7–38.7)	37.4 (34.0–41.0)	34.6 (31.2–38.1)
Parents of Children Ages 6–10								
Child Characteristics								
Total	500	39.3 (34.5–44.3)	28.5 (24.2–33.2)	32.6 (28.1–37.5)	35.2 (30.6–40.2)	32.6 (28.1–37.5)	36.1 (31.5–41.1)	33.0 (28.5–37.8)
Gender								
Male	265	42.5 (35.8–49.6)	30.0 (23.9–36.8)	35.9 (29.4–42.9)	38.9 (32.3–46.0)	34.6 (28.2–41.5)	38.9 (32.3–46.0)	35.8 (29.4–42.8)
Female	228	36.7 (30.0–43.9)	27.5 (21.5–34.4)	29.8 (23.5–36.8)	32.1 (25.6–39.2)	31.3 (24.9–38.3)	34.1 (27.5–41.3)	30.7 (24.4–37.7)
School status								
In-person	124	27.5 (19.6–37.1)	16.7 (10.6–25.2)	21.6 (14.7–30.6)	23.4 (16.0–32.8)	22.6 (15.5–31.7)	24.5 (17.0–33.8)	25.5 (17.8–35.0)
Remote	243	47.3 (40.2–54.5)	37.7 (31.0–44.9)	39.8 (33.0–47.0)	44.5 (37.5–51.7)	39.4 (32.6–46.6)	44.6 (37.6–51.9)	38.4 (31.7–45.6)
Hybrid	132	34.5 (25.9–44.4)	21.3 (14.4–30.3)	28.8 (20.8–38.5)	28.0 (20.1–37.6)	28.9 (20.8–38.5)	30.4 (22.1–40.0)	29.5 (21.4–39.2)
Parent Characteristics								
Gender								
Male	203	35.7 (28.6–43.6)	27.1 (20.7–34.6)	32.9 (26.0–40.7)	32.9 (25.9–40.7)	30.9 (24.2–38.7)	33.9 (26.9–41.8)	32.1 (25.2–40.0)
Female	297	41.8 (35.5–48.3)	29.5 (23.9–35.8)	32.3 (26.5–38.7)	36.9 (30.9–43.4)	33.8 (28.0–40.2)	37.7 (31.6–44.2)	33.6 (27.8–40.0)
Work Status								
Full time^a^	246	38.4 (31.7–45.5)	27.5 (21.6–34.3)	32.8 (26.4–39.8)	34.3 (27.9–41.4)	31.2 (25.0–38.1)	34.3 (27.8–41.3)	33.4 (27.1–40.5)
Part-time	84	42.6 (30.7–55.4)	36.1 (24.9–49.0)	39.2 (27.7–52.1)	36.1 (25.0–49.0)	33.5 (22.8–46.2)	40.6 (28.9–53.4)	34.5 (23.7–47.2)
Not working	170	39.0 (31.0–47.7)	26.8 (19.9–35.0)	29.6 (22.4–38.1)	36.1 (28.3–44.7)	34.2 (26.6–42.8)	36.8 (29.0–45.5)	31.7 (24.3–40.2)
Work at home								
Always	126	53.3 (43.3–63.0)	39.5 (30.2–49.6)	46.8 (37.1–56.8)	45.4 (35.7–55.4)	39.2 (30.0–49.3)	48.5 (38.7–58.5)	45.8 (36.0–55.8)
Sometimes	77	45.0 (32.3–58.3)	36.7 (25.0–50.2)	41.6 (29.2–55.1)	41.2 (28.9–54.8)	38.9 (26.9–52.3)	39.1 (27.1–52.6)	38.1 (26.3–51.4)
No	127	22.0 (14.9–31.3)	15.3 (9.5–23.7)	17.3 (11.1–26.0)	20.3 (13.4–29.6)	20.1 (13.2–29.4)	20.7 (13.8–30.0)	18.8 (12.2–27.9)
Education								
≤ High school	170	31.9 (24.5–40.3)	24.0 (17.4–32.0)	26.7 (19.8–34.9)	28.8 (21.7–37.1)	26.2 (19.4–34.4)	28.4 (21.3–36.7)	26.8 (19.9–35.0)
Some college	156	38.5 (30.1–47.7)	27.6 (20.2–36.4)	29.6 (21.9–38.5)	34.7 (26.6–43.8)	34.3 (26.2–43.4)	37.1 (28.7–46.3)	28.5 (21.0–37.4)
4-year college	115	48.8 (38.2–59.5)	38.0 (28.2–48.9)	43.5 (33.2–54.3)	46.2 (35.8–57.0)	39.8 (29.8–50.6)	44.4 (34.1–55.2)	44.4 (34.1–55.2)
Post-graduate	59	47.9 (33.7–62.4)	28.8 (17.9–42.9)	39.6 (26.6–54.2)	37.9 (25.1–52.6)	36.7 (24.3–51.1)	44.3 (30.5–58.9)	43.2 (29.6–57.9)
Urbanicity								
Big city	102	56.8 (45.4–67.5)	46.6 (35.8–57.8)	50.0 (38.9–61.1)	52.9 (41.6–63.8)	45.7 (34.9–56.9)	49.9 (38.8–61.0)	44.9 (34.1–56.1)
Smaller city	81	42.5 (30.5–55.4)	31.9 (21.2–44.8)	33.5 (22.7–46.4)	34.9 (23.7–48.1)	34.5 (23.4–47.7)	41.0 (29.1–54.0)	32.3 (21.7–45.2)
Suburban	159	37.9 (29.8–46.8)	23.3 (16.7–31.6)	30.9 (23.3–39.7)	35.3 (27.4–44.2)	32.0 (24.4–40.7)	33.9 (26.0–42.7)	33.0 (25.2–41.8)
Small town	72	20.5 (11.7–33.5)	17.8 (9.5–30.8)	18.7 (10.2–31.7)	16.1 (8.4–28.6)	17.9 (9.9–30.2)	19.4 (10.8–32.4)	17.9 (9.9–30.2)
Rural area	85	34.3 (23.6–47.0)	22.8 (14.0–35.0)	26.2 (16.7–38.5)	30.9 (20.7–43.5)	29.5 (19.4–42.0)	34.3 (23.6–47.0)	32.6 (22.1–45.2)
Parents of Youth Ages 11–17								
Child Characteristics								
Total	500	45.4 (40.4–50.6)	34.3 (29.6–39.4)	32.2 (27.6–37.2)	38.4 (33.5–43.6)	37.3 (32.4–42.4)	41.4 (36.4–46.6)	36.4 (31.6–41.5)
Gender								
Male	252	43.1 (36.2–50.3)	33.9 (27.4–41.1)	32.0 (25.7–39.1)	37.0 (30.3–44.2)	36.4 (29.8–43.6)	39.4 (32.6–46.6)	37.3 (30.6–44.6)
Female	239	46.9 (39.4–54.4)	34.4 (27.5–42.0)	31.9 (25.3–39.4)	39.3 (32.1–46.9)	37.5 (30.5–45.1)	43.1 (35.8–50.8)	34.5 (27.7–42.0)
Non-binary	5	73.0 (12.0–98.2)	40.7 (2.7–94.4)	41.2 (2.8–94.5)	41.2 (2.8–94.5)	41.2 (2.8–94.5)	41.2 (2.8–94.5)	41.2 (2.8–94.5)
School status								
In-person	96	34.1 (23.9–46.0)	23.5 (14.9–35.0)	26.2 (17.1–37.9)	28.9 (19.3–40.7)	29.5 (19.9–41.3)	30.5 (20.8–42.4)	30.4 (20.6–42.3)
Remote	251	48.1 (40.9–55.4)	37.2 (30.5–44.6)	32.9 (26.5–40.1)	40.0 (33.1–47.3)	38.4 (31.6–45.7)	43.3 (36.3–50.7)	36.5 (29.8–43.7)
Hybrid	153	47.7 (38.6–57.0)	36.0 (27.4–45.6)	34.6 (26.2–44.0)	41.4 (32.5–50.9)	40.0 (31.2–49.4)	44.6 (35.6–54.0)	39.7 (31.0–49.2)
Parent Characteristics								
Gender								
Male	237	52.6 (39.8–55.2)	40.9 (33.4–48.8)	37.4 (30.2–45.2)	42.0 (34.5–49.9)	42.2 (34.7–50.1)	47.9 (40.2–55.7)	40.6 (33.2–48.5)
Female	263	40.1 (33.6–47.0)	29.4 (23.5–36.1)	28.4 (22.5–35.0)	35.7 (29.4–42.6)	33.6 (27.4–40.4)	36.5 (30.2–43.4)	33.2 (27.1–40.0)
Work Status								
Full time^a^	267	48.7 (41.8–55.7)	37.9 (31.3–44.9)	35.5 (29.1–42.5)	40.0 (33.3–47.1)	38.4 (31.8–45.5)	45.2 (38.4–52.2)	39.1 (32.5–46.2)
Part-time	55	41.8 (27.6–57.4)	28.5 (16.7–44.2)	29.3 (17.3–45.1)	36.6 (23.1–52.6)	31.8 (19.5–47.4)	36.1 (22.6–52.2)	30.6 (18.5–46.1)
Not working	178	41.5 (33.2–50.4)	30.7 (23.1–39.5)	28.1 (20.8–36.7)	36.5 (28.4–45.3)	37.2 (29.1–46.1)	37.2 (29.1–46.0)	34.0 (26.2–42.8)
Work at home								
Always	114	53.9 (43.5–63.9)	40.0 (30.3–50.5)	39.8 (30.2–50.3)	46.4 (36.3–56.8)	44.3 (34.3–54.7)	49.8 (39.5–60.1)	44.2 (34.2–54.7)
Sometimes	68	54.5 (40.5–67.8)	42.8 (29.5–57.2)	40.0 (27.0–54.5)	43.1 (29.9–57.5)	37.4 (24.9–52.0)	49.7 (35.9–63.5)	39.3 (26.4–53.8)
No	140	37.5 (28.4–47.6)	29.1 (20.8–39.1)	26.2 (18.5–35.8)	30.7 (22.1–40.8)	30.6 (22.1–40.7)	34.5 (25.7–44.6)	30.5 (22.1–40.5)
Education								
≤ High school	152	45.6 (36.6–54.9)	36.7 (28.2–46.1)	35.0 (26.7–44.4)	40.5 (31.8–50.0)	39.7 (31.1–49.1)	41.8 (33.0–51.2)	36.7 (28.3–46.1)
Some college	167	45.8 (36.7–55.2)	34.5 (25.9–44.1)	26.9 (19.3–36.1)	39.0 (30.3–48.5)	36.9 (28.3–46.4)	40.9 (32.0–50.4)	37.3 (28.7–46.8)
4-year college	109	41.1 (31.3–51.7)	28.0 (19.5–38.5)	28.4 (20.0–38.7)	33.5 (24.3–44.2)	31.8 (22.9–42.3)	39.4 (29.6–50.0)	32.7 (23.6–43.3)
Post-graduate	72	52.2 (38.9–65.2)	40.0 (27.7–53.6)	41.8 (29.4–55.3)	41.2 (28.8–54.8)	42.2 (29.7–55.8)	45.0 (32.2–58.4)	40.6 (28.3–54.2)
Urbanicity								
Big city	93	66.5 (54.5–76.7)	52.8 (41.1–64.2)	47.6 (36.2–59.2)	53.1 (41.4–64.5)	57.6 (45.8–68.7)	60.7 (48.8–71.4)	53.1 (41.4–64.5)
Smaller city	78	45.6 (32.6–59.2)	27.7 (16.7–42.3)	27.3 (16.5–41.6)	41.8 (29.0–55.7)	33.8 (21.9–48.1)	38.9 (32.5–49.5)	35.4 (23.3–49.7)
Suburban	177	42.5 (34.2–51.2)	32.6 (24.9–41.3)	32.1 (24.5–40.7)	36.6 (28.6–45.4)	33.3 (25.7–41.9)	40.7 (32.5–49.5)	34.1 (26.4–42.7)
Small town	68	34.6 (22.8–48.8)	26.6 (16.1–40.7)	20.9 (11.7–34.4)	24.5 (14.4–38.4)	26.0 (15.6–40.2)	26.4 (16.0–40.4)	25.4 (15.3–39.1)
Rural area	84	34.1 (22.8–47.6)	27.5 (17.2–40.9)	27.3 (17.0–40.8)	32.3 (21.2–45.9)	33.4 (22.2–46.9)	33.4 (22.2–46.9)	30.6 (19.7–44.2)

*PA* physical activity, *CI* confidence intervals

^a^Over 35 h/week

Among parents of younger children, parents whose children were attending school remotely were more likely to have interest in physical activity information, compared to those whose children were attending school in-person and whose school was hybrid (both remote and in-person). For example, significantly more remote school parents reported interest in information [47.3% (95% CI: 40.2–54.5%)], compared to parents whose children were attending school in-person [27.5% (95% CI: 19.6–37.1%); *p* < 0.001] and parents whose child’s school was hybrid [34.5% (95% CI: 25.9–44.4%); *p* = 0.02]. Differences in information needs were also seen by parents’ working from home status. Among parent participants who worked, less than a quarter of parents who were working outside the home had interest in information on physical activity [22.0% (95% CI: 14.9–31.3%)], compared to parents who sometimes [45.0% (95% CI: 32.3–58.3%); *p* < 0.001] or always [53.3% (95% CI: 43.3–63.0%); *p <* 0.001] worked from home.

There were also differences in reported parent information needs by urbanicity level. Among parents of younger children, 56.8% (95% CI: 45.4–67.5%) of parents living in a big city had interest in physical activity information, compared to 37.9% (95% CI: 29.8–46.8%) in a suburban areas (*p* = 0.003), 20.5% (95% CI: 11.7–33.5%) in small towns (*p* < 0.001), and 34.3% (95% CI: 23.6–47.0%) in rural areas (*p* = 0.002). Similar differences by urbanicity were seen among parents of older children. Nearly two-thirds (66.5%; 95% CI: 54.5–76.7%) of parents living in a big city had interest in information, compared to 45.6% (95% CI: 32.6–59.2%) in smaller cities (*p* = 0.006), 42.5% (95% CI: 34.2–51.2%) in suburban areas (*p* < 0.001), 34.6% (95% CI: 22.8–48.8%; *p <* 0.001) in small towns (*p <* 0.001), and 34.1% (95% CI: 22.8–47.6%) in rural areas (*p <* 0.001).

### Child information needs

Child-reported physical activity information needs, examined among older children only, were similar overall to parent information needs. For example, 41.5% (95% CI: 36.5–46.7%) of children had interest in information about being active during COVID-19, compared to 41.9% (95% CI: 38.9–45.5%) of parents (Table 3). Regarding the kind of information children wanted, as with parents, interest tended to be lower among COVID-19-specific information and higher for online physical activity resources and physical activity ideas they could do alone, inside, or outdoors.

**Table 3:** Child Physical Activity Information Needs by Child and Parent Characteristics among Children ages 11–17

		Interest in information about being active during COVID-19 (% Yes)	“What kind of information would you want?” (% Yes)					
			Why it’s important to be active during COVID-19	How to avoid getting COVID-19 while doing PA	Online resources for doing PA	Ideas about PA you can do…		
						…alone	…inside	…outdoors
	n	% (95% CI)	% (95% CI)	% (95% CI)	% (95% CI)	% (95% CI)	% (95% CI)	% (95% CI)
Total	500	41.5 (36.5–46.7)	28.1 (23.7–33.1)	28.7 (24.2–33.6)	32.4 (27.8–37.5)	36.1 (31.3–41.2)	36.0 (31.2–41.1)	33.9 (29.2–39.0)
Child Characteristics								
Gender								
Male	252	42.0 (35.2–49.2)	28.1 (22.1–35.0)	28.7 (22.6–35.6)	31.7 (25.5–38.7)	36.0 (29.4–43.8)	37.0 (30.4–44.1)	33.1 (26.7–40.1)
Female	239	41.1 (33.9–48.7)	27.8 (21.5–35.3)	28.3 (21.9–35.7)	33.0 (26.2–40.7)	36.1 (29.1–43.8)	35.6 (28.7–43.2)	34.7 (27.8–42.3)
Non-binary	5	41.2 (2.8–94.5)	41.2 (2.8–94.5)	41.2 (2.8–94.5)	41.2 (2.8–94.5)	41.2 (2.8–94.5)	9.0 (0.3–77.1)	41.2 (2.8–94.5)
School status								
In-person	96	34.5 (24.1–46.6)	16.9 (9.6–28.0)	17.7 (10.4–28.5)	27.0 (17.7–38.9)	28.5 (18.8–40.6)	32.9 (22.8–45.0)	29.9 (20.1–42.0)
Remote	251	42.7 (35.7–50.0)	30.8 (24.5–37.9)	31.5 (25.2–38.6)	32.6 (26.1–39.7)	36.6 (29.9–43.9)	36.2 (29.5–43.5)	36.0 (29.3–43.2)
Hybrid	153	43.7 (34.8–53.1)	30.5 (22.4–39.9)	30.6 (22.5–40.1)	35.4 (27.0–44.9)	39.7 (30.9–49.1)	37.5 (29.0–47.0)	33.0 (24.8–42.4)
PA barriers								
Don’t like doing PA	186	33.6 (26.0–42.1)	23.3 (16.8–31.4)	22.9 (16.5–31.0)	26.9 (19.9–35.3)	32.1 (24.6–40.6)	28.5 (21.4–37.0)	27.4 (20.4–35.7)
Don’t know what to do	179	41.9 (33.7–50.4)	27.2 (20.2–35.6)	28.4 (21.3–36.8)	31.8 (24.4–40.3)	35.6 (27.9–44.1)	36.8 (29.0–45.4)	35.2 (27.5–43.8)
It’s not safe	111	55.2 (44.4–65.6)	50.2 (39.5–60.9)	50.3 (39.6–61.0)	45.2 (34.7–56.1)	46.5 (36.0–57.4)	50.5 (39.7–61.1)	45.0 (34.5–55.9)
No one to play with	164	46.6 (37.6–55.8)	32.6 (24.4–42.1)	33.2 (24.9–42.5)	37.6 (29.1–47.0)	40.8 (32.1–50.2)	39.1 (30.4–48.4)	35.8 (27.4–45.1)
Weather not good	238	44.9 (37.7–52.3)	32.0 (25.4–39.5)	32.7 (26.1–40.2)	40.7 (33.6–48.2)	40.6 (33.5–48.1)	41.7 (34.6–49.2)	37.9 (30.9–45.3)
No equipment	121	48.4 (38.4–58.6)	37.3 (27.9–47.7)	35.6 (26.4–46.1)	41.3 (31.7–51.6)	42.2 (32.5–52.5)	45.9 (36.0–56.2)	37.0 (27.7–47.4)
Worried about COVID	192	57.8 (49.5–65.6)	44.9 (36.9–53.2)	47.4 (39.4–55.6)	46.7 (38.7–55.0)	51.2 (43.1–59.3)	50.7 (42.6–58.9)	47.1 (39.0–55.3)
Can’t access online resources	71	65.3 (51.8–76.7)	54.2 (40.9–67.0)	57.3 (43.9–69.7)	54.2 (40.8–67.0)	53.1 (39.8–66.0)	57.2 (43.8–69.6)	56.6 (43.2–69.1)
Parent Characteristics								
Gender								
Male	237	50.5 (42.8–58.2)	34.2 (27.1–42.1)	32.9 (25.8–40.8)	39.1 (31.7–47.1)	43.0 (35.5–51.0)	43.7 (36.1–51.6)	41.4 (33.9–49.4)
Female	263	34.8 (28.6–41.5)	23.6 (18.3–29.9)	25.6 (20.1–31.9)	27.5 (21.8–34.0)	30.9 (25.0–37.5)	30.3 (24.5–36.9)	28.4 (22.6–34.9)
Work Status								
Work full time^a^	267	48.6 (41.7–55.6)	32.3 (25.9–39.3)	35.5 (28.9–42.6)	38.0 (31.4–45.2)	40.7 (33.9–47.8)	41.3 (34.5–48.4)	40.4 (33.7–47.5)
Work part-time	55	39.0 (25.1–54.9)	27.2 (16.0–42.3)	25.6 (14.6–41.0)	33.2 (20.4–49.1)	39.0 (25.1–54.9)	34.4 (21.1–50.7)	57.4 (15.9–43.2)
Not working	178	31.5 (24.1–39.9)	22.2 (15.9–30.1)	19.3 (13.5–26.8)	23.7 (17.2–31.8)	28.3 (21.2–36.5)	28.5 (21.6–36.7)	26.0 (19.1–34.2)
Working from home								
Yes, all the time	114	50.3 (40.1–60.6)	35.0 (25.7–45.6)	41.2 (31.4–51.8)	38.9 (29.3–49.5)	40.2 (30.5–50.8)	44.4 (34.3–54.9)	42.2 (32.3–52.7)
Yes, sometimes	68	60.1 (46.0–72.6)	42.4 (29.2–56.8)	36.9 (24.4–51.4)	45.5 (32.1–59.7)	52.7 (38.8–66.2)	48.1 (34.4–62.1)	47.9 (34.2–61.9)
No	140	36.3 (27.2–46.5)	21.6 (14.3–31.5)	25.0 (17.1–35.1)	30.8 (22.2–41.0)	33.4 (24.6–43.6)	31.5 (22.9–41.6)	29.0 (20.7–39.0)
Parent Education								
≤ High school	152	34.1 (26.0–43.3)	25.7 (18.5–34.5)	26.5 (19.2–35.3)	29.0 (21.4–38.1)	31.9 (24.0–41.1)	33.0 (25.0–42.2)	29.2 (21.6–38.1)
Some college	167	41.8 (32.8–51.3)	30.1 (22.1–39.6)	25.8 (18.4–35.0)	32.9 (24.1–42.0)	38.1 (29.5–47.6)	37.5 (28.9–47.0)	32.1 (23.9–41.6)
4-year college	109	42.2 (32.3–52.9)	25.4 (17.1–36.0)	28.9 (20.2–39.4)	32.7 (23.5–43.1)	36.9 (27.3–47.6)	34.5 (25.2–45.1)	33.2 (24.0–43.9)
Post-graduate	72	56.9 (43.5–69.4)	35.4 (23.7–49.2)	38.5 (26.3–52.3)	40.3 (28.0–54.1)	40.8 (28.4–54.4)	43.1 (30.4–56.7)	49.4 (36.3–62.6)
Urbanicity								
Big city	93	57.2 (45.3–68.3)	48.8 (37.3–60.4)	47.3 (35.9–58.9)	47.9 (36.5–59.5)	44.1 (33.0–55.8)	51.1 (39.4–62.5)	46.8 (35.5–58.5)
Smaller city	78	36.9 (24.8–50.8)	22.6 (13.0–36.4)	26.1 (15.9–39.8)	31.2 (20.0–45.2)	36.4 (24.4–50.4)	26.2 (16.1–39.7)	27.9 (17.3–41.6)
Suburban	177	43.5 (35.1–52.2)	28.4 (21.0–37.1)	30.2 (22.8–38.9)	32.1 (24.5–40.8)	38.0 (30.0–46.8)	39.0 (30.9–47.8)	35.5 (27.6–44.2)
Small town	68	33.4 (21.8–47.5)	17.7 (9.2–31.4)	19.2 (10.3–33.0)	26.9 (16.3–41.0)	31.3 (20.0–45.3)	29.5 (18.5–43.6)	30.1 (19.0–44.2)
Rural area	84	27.8 (17.8–40.7)	15.1 (8.2–26.2)	11.5 (5.9–21.5)	19.1 (10.6–31.8)	25.0 (15.4–38.0)	24.7 (15.3–37.2)	22.6 (13.4–35.6)

*PA* physical activity, *CI* confidence intervals

^a^Over 35 h/week

Children whose parents worked full-time were more likely to have interest in physical activity information. For example, 48.6% (95% CI: 41.7–55.6%) of youth whose parents worked full-time were interested, compared to 31.5% (95% CI: 24.1–39.9%) of youth whose parents did not work (*p* < 0.001). Differences by urbanicity level were also seen, with children from a big city being more likely to report interest in physical activity information than those from other areas. For example, 57.2% (95% CI: 45.3–68.3%) of children living in a big city were interested, compared to 36.9% (95% CI: 24.8–50.8%) in smaller cities (*p* = 0.008), 43.5% (95% CI: 35.1–52.2%) in suburban areas (*p* = 0.03), 33.4% (95% CI: 21.8–46.5%) in small towns (*p* = 0.003), and 27.8% (95% CI: 17.8–40.7%) in rural areas (*p* < 0.001).

Children’s interest in physical activity information varied by reported barriers. Interest in information was highest among children who wanted to do online physical activity but did not have the right resources (65.3%; 95% CI: 51.8–76.7%), followed by those who were worried about getting COVID-19 (57%; 95% CI: 49.5–65.6%) and those who reported that it was not safe to go outside their home to be active (55.2%; 95% CI: 44.4–65.6%).

## Discussion

This study among a nationally representative sample found that over 40% of parents of children 6 to 17 years-old were interested in receiving information about helping their family be active during the COVID-19 pandemic. Parents who were more likely to be interested in information were those working from home compared to outside the home, living in a big city compared to a suburban area, small town, or rural area, and those whose children were doing school remotely compared to in-person. As with parents, 41.5% of children 11–17 years-old were interested in information about being active during the pandemic. Children more likely to be interested in information were those whose parents worked full-time compared to those not working, and those living in a big city compared to a rural area.

Parents of younger children who were attending school remotely were more likely to be interested in information about helping their family be active during COVID-19, compared to those whose children were attending school in-person. Schools typically play an important role in supporting student activity by providing opportunities such as PE, recess, and classroom movement opportunities [31]. However, unique challenges arise with remote learning compared to in-person learning, including adapted PE lessons and classroom activity, lack of recess, and disruptions to or cancellation of school-based activities. Therefore, parents with children who are attending school remotely may be taking on a larger responsibility for their child’s daily physical activity behavior during the pandemic and in search of resources to help keep their family active. Additionally, children are likely spending more time sedentary with remote learning, which may further prompt parents to seek additional support for keeping their children active. It is also of note that differences in information needs by school status (remote, in-person, hybrid) were greater among parents of younger children compared to those with older children, which may be due to the lower autonomy and greater parent involvement among younger children. Overall, schools offering remote learning can support families in promoting physical activity by sharing resources and encouraging family-centered physical activity when possible. The Comprehensive School Physical Activity Program (CSPAP), a multi-component approach for schools to support student activity [32], can be utilized to identify opportunities for increasing family activity while children are attending school remotely. Further, strategies for increasing family-engaged activity during the pandemic (e.g., supporting active physical activity classroom movement breaks, access to school playgrounds and fields), can potentially be beneficial for CSPAP-consistent physical activity post-pandemic.

Parents working from home were more likely to be interested in physical activity information compared to parents working outside of the home. As with remote learning, parents who are spending more time at home may be taking on a larger role in their families’ daily activity, need to keep their children occupied while parents work from home, and/or are observing children’s lack of activity more and therefore are more likely to be interested in resources that help facilitate healthy behaviors. Another possibility is that families in which parents are working from home are experiencing more disruptions to organized opportunities for physical activity. For example, areas in which businesses are encouraging employees to work from home may also have more restrictions due to the pandemic including park closures, cancellation of organized sports, and transitions to remote learning. Efforts to address children’s activity levels during the pandemic should capitalize on the overall interest in physical activity-related information among parents, particularly those working from home and who have children attending school virtually.

Children with parents working full-time were more likely to be interested in information about being active during the pandemic compared to those whose parents were not working. One explanation is that if parents are spending a majority of their time working, older children may be interested in identifying ways to stay active, independent of their parents. Physical activity resources are particularly relevant for youth whose usual means for staying active (e.g., school activity, organized sport) have been disrupted due to the pandemic. For parents working full-time pre-pandemic, their children may be more likely to have been enrolled in organized activities – in part as a form of child supervision – while parents worked. With a majority of these activities being canceled in response to the pandemic, these children likely have a greater change in their physical activity levels. It is also of note that the parents and youth with interest in information about staying active during the pandemic did not have these needs met from other sources. While schools, community organizations, and sport and recreation programs are adapting to their new reality in various ways, there are opportunities to meet families’ needs around physical activity.

Parents and children living in big cities were more likely to report interest in information about staying active during COVID-19, compared to those living in small towns or rural areas. A potential explanation may be the varying degree of restrictions and closures that were imposed across the US in response to the pandemic. The authority for imposing COVID-19 restrictions, including sheltering in place orders and school or business closures, rests with states and localities. Thus, when and to what extent COVID-19 restrictions are implemented varies at the local level, with more restrictions often seen among high-population density cities [33]. Fewer restrictions and closures of parks, businesses, and schools means less disruption in low-population density areas such as small cities and rural areas, which may account for the lower interest in information among parents and children from these areas. In areas where opportunities for physical activity (parks, schools, etc.) remain accessible to families, there is likely to be less demand for information regarding how to stay active during the pandemic. This is further supported by evidence suggesting that time spent at home since the start of the pandemic has minimally increased for those in rural areas or small towns compared to those in big cities [34], and a larger decrease in physical activity levels among adolescents in urban compared to rural areas [35].

When considering children’s reported barriers to physical activity, interest in information about being active was highest among children who wanted to do online physical activity but did not have the right resources (e.g., laptop, Wi-Fi), children who were worried that they might get COVID-19, and children who reported that it was not safe to go outside near their home to play or be active. Further, within each of these groups, almost or over half of children reported interest across all the different types of information (e.g., how to avoid getting COVID-19 while doing physical activity; online resources for doing physical activity; ideas for physical activity you can do alone, inside, outdoors; etc.). These findings have implications for the type of information that should be provided to children to help them feel confident in their ability to be physically active during the pandemic, such as how to safely participate in physical activity both within and outside their home. It is also important to ensure that resources are developed and delivered equitably, such that information is accessible to youth regardless of their access to information and communication technologies (e.g., digital divide).

Addressing physical activity-related information needs can help to support family and youth participation in physical activity during and after the pandemic. In addition to the well-established physical and mental health benefits of physical activity, [4] consistently meeting physical activity guidelines has been strongly associated with a reduced risk for severe COVID-19 among infected adults [36]. Further, there is evidence that physical activity may be protective against the negative impact of pandemic-related stressors and support positive mental health in both adult and youth populations [37–39]. Notably, the issue of physical inactivity among youth has been further exacerbated by the pandemic, and the need for identifying how to equitably support physical activity among youth and their families is critical.

### Limitations and strengths

This study was conducted among a large, nationally representative sample of parents and children, which provides external study validity and the ability to examine differences in information needs by sociodemographic variables. This is also the first known study to examine physical activity-related information needs among parents and children during the COVID-19 pandemic, which can inform educators, community organizations, and researchers in developing resources for families. This is an important first step in developing interventions that take families felt needs into account and highlights how differences in information needs should be taken into consideration to efficiently support families’ physical activity engagement during and after the pandemic. These findings can also assist practitioners and health care providers in meeting their patients’ specific physical activity informational needs, promoting increased quality of care [40]. The limitations of this study must also be noted. First, these data are self-report and therefore subject to recall bias and social desirability bias. Second, this survey was conducted in Fall 2020 and pandemic-related circumstances may have changed over time, such as the status of schools, sports, and other opportunities for physical activity. Third, although YouGov’s sample selection using matching methodology allows for selection of representative samples in terms of the proportion of respondents from key demographic characteristics, responses may not be representative.

## Conclusions

Overall, parents and children are interested in information about staying active during COVID-19, which presents novel barriers to physical activity engagement. Families most likely to be interested in these resources are those whose daily routines and opportunities for physical activity may have been most significantly impacted by the pandemic, including parents who always worked from home and whose children attended school remotely. Children most interested in information were those who did not have the right resources to engage in online physical activity or those who were worried about their safety or getting infected with COVID-19. Identifying felt needs is an important step in developing tailored interventions that aim to effectively and sustainably support families in promoting physical activity. Behavioral interventions oriented around increasing youth physical activity should provide guidance that resonates with families and accounts for setting-specific constraints and stressors.

## Data Availability

The datasets used and/or analyzed during the current study are available from the corresponding author on reasonable request.

## Abbreviations

SES: Socioeconomic status
PE: Physical education
ACS: American Community Survey
IRB: Institutional review board
SD: Standard deviation
CI: Confidence interval
CSPAP: Comprehensive School Physical Activity Program
